# A Portable Fluorescent Lateral Flow Immunoassay Platform for Rapid Detection of FluA

**DOI:** 10.3390/bios14060263

**Published:** 2024-05-21

**Authors:** Xu Chen, Xuhui Huang, Saima Kanwal, Jian Wang, Jing Wen, Dawei Zhang

**Affiliations:** 1 School of Optical-Electrical and Computer Engineering, University of Shanghai for Science and Technology, Shanghai 200093, China; 191380026@st.usst.edu.cn (X.C.); huangxuhui1213@163.com (X.H.); saima@usst.edu.cn (S.K.); jian_wang@fpi-inc.com (J.W.); dwzhang@usst.edu.cn (D.Z.); 2 Engineering Research Center of Optical Instrument and System, The Ministry of Education, Shanghai Key Laboratory of Modern Optical System, University of Shanghai for Science and Technology, Shanghai 200093, China

**Keywords:** lateral flow immunoassay, quantum-dot fluorescent microspheres, optical pick-up unit, FluA, point-of-care

## Abstract

The spread of the FluA virus poses significant public health concerns worldwide. Fluorescent lateral flow immunoassay (LFIA) test strips have emerged as vital tools for the early detection and monitoring of influenza infections. However, existing quantitative virus-detection methods, particularly those utilizing smartphone-based sensing platforms, encounter accessibility challenges in resource-limited areas and among the elderly population. Despite their advantages in speed and portability, these platforms often lack user-friendliness for these demographics, impeding their widespread utilization. To address these challenges, this study proposes leveraging the optical pick-up unit (OPU) sourced from commercial optical drives as a readily available fluorescence excitation module for the quantitative detection of antibodies labeled with quantum-dot fluorescent microspheres. Additionally, we utilize miniaturized and high-performance optical components and 3D-printed parts, along with a customized control system, to develop an affordable point-of-care testing (POCT) device. Within the system, a stepping motor scans the test strip from the T-line to the C-line, enabling the calculation of the fluorescence-intensity ratio between the two lines. This simple yet effective design facilitates rapid and straightforward field or at-home testing for FluA. The proposed prototype platform demonstrates promising performance, achieving a limit of detection (LOD) of 2.91 ng/mL, a total detection time of no more than 15 min, and dimensions of 151 mm × 11.2 mm × 10.8 mm^3^. We believe that the proposed approach holds great potential for improving access to an accurate influenza diagnosis.

## 1. Introduction

Preventing and monitoring infectious diseases are pivotal to safeguarding public health [1,2]. The recurrent prevalence of influenza A (FluA) raises substantial public health concerns, especially for vulnerable populations such as the elderly and children. As a matter of course, there is a greatly urgent demand for convenient, fast, and cost-effective diagnostic tools for real-time detection that can be used on a large scale [3].

Currently, various molecular diagnostic methods, such as enzyme-linked immunosorbent assay (ELISA) [4], polymerase chain reaction (PCR) [5], and microarray chips [6], are utilized for their high accuracy. However, these methods necessitate specialized equipment and skilled personnel, making rapid self-testing challenging during outbreaks. Hence, during influenza outbreaks, there is a high demand for point-of-care testing (POCT) devices, especially in resource-limited regions, to mitigate morbidity and mortality [7].

In the past decades, lateral flow immunochromatography (LFIA) has emerged as a pivotal tool in bioanalysis, known for its simplicity, rapidity, affordability, and sensitivity. Moreover, LFIA offers the advantage of high specificity in the interaction between the target and the receptor. Artificially designed or naturally occurring probes are specifically combined with key biochemical markers and then read out by optical, thermal, magnetic, or electrochemical methods [8]. At present, LFIA has been widely applied as a promising molecular detection assay in clinical diagnosis [9], pharmaceutical testing [10], food contamination and residue testing [11], and environmental monitoring [12]. Immunochromatographic test strips (ICTS) represent the most common form of POCT device based on LFIA technology. These strips serve as carriers for immune reactions, facilitating both qualitative and quantitative molecular detection [13].

To date, numerous studies have highlighted the advances of POCT devices based on ICTS, enabling rapid, in-vitro, low-cost, and on-site analysis of biochemical substances [2,3,9,14,15,16,17]. However, traditional colorimetric analysis-based LFIA suffers from limited detection sensitivity and qualitative or semi-quantitative detection capabilities [17]. The widespread use of fluorescent markers has spurred the development of fluorescence-sensing-based LFIA, utilizing the unique properties of materials such as carbon dots (CDs) [18], quantum dots (QDs) [8], up-conversion nanoparticles [19], and time-resolved fluorescent microspheres [20] for detection purposes. These materials generate specific fluorescent signals during the detection process, enabling precise detection and quantitative analysis of the target substance through signal detection and analysis. Notably, the integration of QDs with polymer microspheres has led to the formation of quantum-dot fluorescent microspheres (QDFM), exhibiting enhanced fluorescence effects and substantially improved detection sensitivity compared to conventional QDs [1,2,13,17].

Meanwhile, the current fluorescence LFIA platform predominantly relies on imaging analysis and optical scanning methods. Imaging-based diagnostic devices have progressively emerged as optimal readout platforms for bioanalytical applications, integrating diverse accessories and customized software solutions. Among these, smartphones have gained prominence as POCT devices due to their portability, high-performance processor, and seamless data transmission [14,15,16,17]. However, the performance of the smartphone cameras limits the analysis sensitivity and resolution. Detecting low concentrations of target substances or distinguishing subtle signal differences requires higher-performing smartphones, thereby increasing system costs. Additionally, discrepancies in camera performance and operating systems among smartphone brands and models hinder universal adoption, impeding the establishment of a standardized detection and analysis framework [21]. Furthermore, uptake in developing countries or resource-constrained areas is hindered by limited smartphone access, complex applications, and individual conditions, particularly among elderly users. As one of the key technologies in the LFIA platform, optical scanning technology can achieve precise movement and positioning of the test strips, thus enhancing the accuracy and reliability of testing.

We considered the requirements of miniaturization, low cost, and replicability of POCT devices. Hence, in this study, we have developed a portable, economical, stand-alone, and user-friendly fluorescent LFIA platform based on an optical pick-up unit (OPU). We utilized a stepping motor scanning mechanism, employed the prepared QDFM-mAb (monoclonal antibody) complex as a fluorescent probe, and used the prepared test paper strip as a carrier for point-of-care detection of FluA. The incorporation of a commercially available OPU as the platform’s fluorescence excitation module underscores its accessibility and mass-produced nature, seamlessly integrated with 3D-printed components, compact optical elements, and customized electronic modules. The OPU unit contains a series of high-performance optoelectronic assemblies, such as a laser diode, laser driver, objective lens, collimating lens, voice coil motor, and so on. These off-the-shelf, high-performance components, compact in design, facilitate the miniaturization of our detection platform while concurrently reducing costs and complexity. Our platform is designed to detect FluA with high sensitivity and specificity. Furthermore, owing to its affordability, portability, and analytical performance, it holds immense potential for rapid and precise POCT of FluA.

## 2. Materials and Methods

### 2.1. Principle of the LFIA Detection Platform

**Figure 1:**
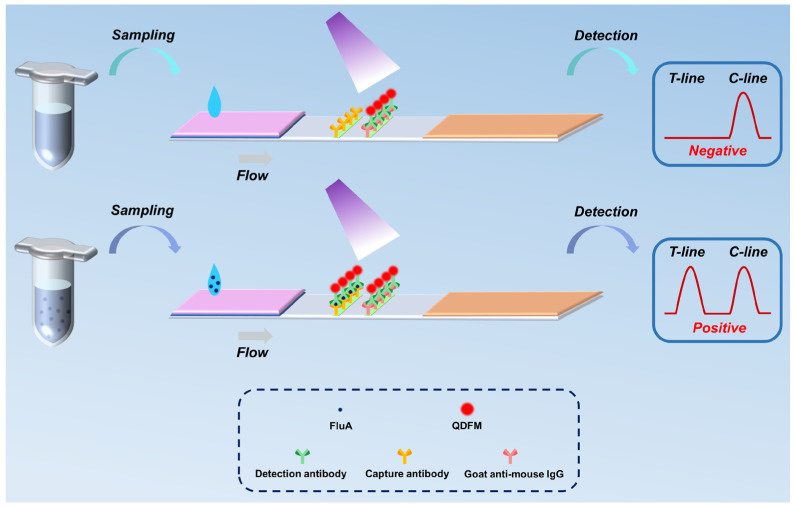
The schematic illustration of the proposed platform for the detection of FluA.

Figure 1 depicts the schematic diagram of the fluorescence LFIA platform for detecting FluA. During the detection process, the sample solution is sampled and loaded onto the ICTS sample pad, migrating towards the absorbent pad due to capillary action. When the sample contains the target FluA virus, the QDFM antibody can recognize and capture the target analyte. Ultimately, through an immunoreaction and binding with the capture antibody, a sandwich immunocomplex is formed and fixed on the T-line. Any surplus QDFM antibody biolinkers continue migrating and are eventually affixed on the C-line. Upon laser excitation, fluorescence signals become visible to the human eye when the target analyte concentration is high. Similarly, in the absence of the target FluA virus in the sample solution, the capture antibody on the T-line remains inactive, yielding solely the fluorescence intensity value on the C-line. In this study, the ICTS is inserted into the detection platform to scan the fluorescence intensities of the T-line and C-line for a further quantitative analysis of the FluA.

### 2.2. Optical Pick-Up Unit (OPU)

**Figure 2:**
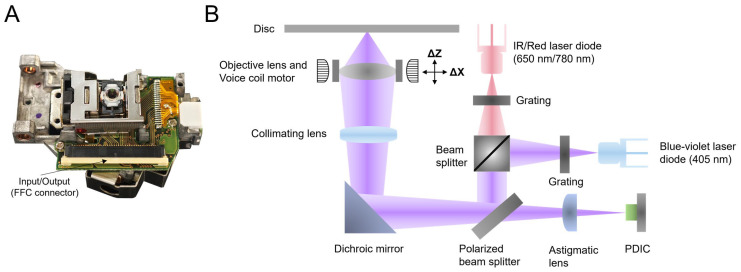
(**A**) The visual representation of the OPU extracted from a commercial HD-DVD drive. External communication is conducted through the flexible flat cable (FFC) handling signal input and output. (**B**) The internal optical path system diagram of the OPU. The red and blue–violet beams represent a laser emitted from a 650 nm/780 nm and a 405 nm laser diode, respectively. (IR: infrared red; PDIC: photodiode integrated circuit).

In the past decades, the OPU has gradually become the focus of research owing to its compact volume, low cost, and high performance. This assembly is widely used in research fields that pursue miniaturization and cost-effectiveness, including but not limited to microscopy [22,23,24], micro-nanomanufacturing [25,26,27], biosensing [28,29,30,31], etc. Particularly in biosensing, the high sensitivity and rapid-response characteristics of the OPU make it an effective tool for detecting biomolecules and cells. Combined with techniques such as fluorescence labeling, the OPU can achieve high-sensitivity detection of the target molecules in biological samples, thus offering crucial support for biomedical research and clinical diagnosis. The visual representation and internal key components of the commercial OPU are depicted in Figure 2, showcasing its compact size, roughly equivalent to a matchbox, and weighing only 22 g, underscoring its lightweight and portable attributes.

In the OPU operation, the laser beam emitted from the 405 nm or 650 nm/780 nm laser diode propagates through the designated optical path, as clearly depicted in Figure 2B. After traversing the beam splitter and mirror, it undergoes collimation via a collimating lens. Subsequently, the beam is focused with utmost efficiency through an objective lens with a numerical aperture (NA) of 0.65 directly onto the sample. Throughout this process, the voice coil motor (VCM) carries the objective lens for nanometer-level motion, and the quadrant photodiode integrated circuit (PDIC) controls the laser beam, ensuring its precise focus on the surface of the disc. Moreover, the OPU features a built-in laser-diode driver, necessitating solely the input of different level voltages to specific pins. The relevant details are available in our previous work [32]. The laser power emitted by the laser diode in the OPU can be adjusted by the pulse width modulation (PWM) signal input from the external control board. More details about the laser diode are in the Supplementary Materials.

### 2.3. Construction of the Platform

The construction of the proposed platform is based on the fluorescence detection principle of QDFM ICTS. To optimize the fluorescence detection efficiency, it is necessary to select suitable components. The platform mainly consists of three parts: an optical detection system, an electronic system, and a casing. 

**Figure 3:**
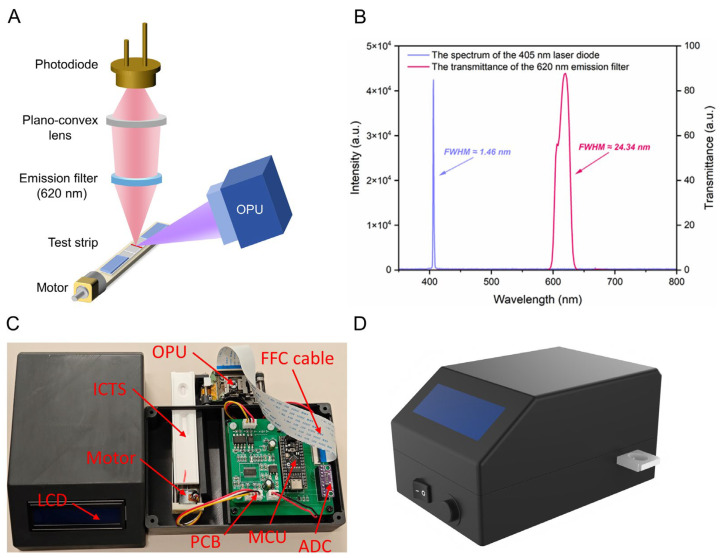
(**A**) Schematic diagram of the fluorescence-detection platform. (**B**) The spectrum of the 405 nm laser diode of the OPU and the transmittance of the 620 nm emission filter. (**C**) Physical drawing of the proposed platform and its sub-components. (**D**) The 3D rendering of the proposed platform. (OPU: optical pick-up unit, ADC: analog-to-digital converter, MCU: microcontroller unit, FFC: flexible flat cable, LCD: liquid crystal display, ICTS: immunochromatographic test strip, PCB: printed circuit board).

The optical detection system is responsible for the excitation and acquisition of the fluorescence signal of the loaded test strip. The system’s design fulfills the criteria of portability and miniaturization while operating with optimal efficiency. Figure 3A illustrates its structure. The optical center of the excitation light-source unit and the fluorescence signal-collection unit of the system converge at a specific point on the test strip. Driven by the stepping motor, the test strip passes through the convergence point. The excitation light-source unit excites the fluorescence markers on the test strip, while the fluorescence signal-collection unit detects the intensity of the fluorescence, completing the scan of the test strip.

#### 2.3.1. Fluorescence Excitation and Detection Module

The fluorescence excitation module consists solely of a commercial OPU (PHR-803T, Toshiba, Tokyo, Japan) and is used to excite the fluorescence markers on the test strip. As illustrated in Figure 3B, the OPU exhibits prominent monochromaticity (full width at half maximum, FWHM ≈ 1.46 nm) for the 405 nm laser and provides a focused spot within the diffraction limit as well. As a matter of fact, under the condition of ensuring power and temperature stability, a laser diode would be a suitable choice, especially in terms of monochromaticity, coherence, and directionality, as well as cost and size. These eliminate the traditional fluorescence-detection device’s reliance on high-performance excitation filters and lenses, significantly reducing the cost, size, and system complexity of the proposed device. The fluorescence signal-acquisition module comprises a plano-convex lens (Grand Unified Optics, Beijing, China), a band-pass emission filter (620 nm, PuxianTec, Shanghai, China), and a photodiode (S1227-1010BQ, Hamamatsu, Tokyo, Japan). The plano-convex lens magnifies the fluorescence spot for signal detection. The transmission curve of the narrowband filter is shown in Figure 3B. Its passband center wavelength is approximately 620 nm, with a FWHM of approximately 24.34 nm and a peak transmittance of 87.48%. Its function is to retain red fluorescence, with a wavelength close to 625 nm, while filtering out other wavelengths of stray light interference. The fluorescence signal is finally collected by a high-sensitivity photodiode, which has a light-receiving area of up to 10 × 10 mm^2^. It exhibits peak sensitivity at 720 nm and a dark current of only 50 pA, which meets our requirements. The optical axis of the entire excitation light path is oriented at a 45° angle concerning the optical axis of the fluorescence-detection light path. This configuration maximizes the measurement of fluorescence signals reflected from the ICTS’s surface. 

#### 2.3.2. Casing Construction

Figure 3C,D shows the appearance and internal structure of the prototype fluorescence-detection platform for ICTS based on OPU. Figure 3C shows the prototype of our proposed platform. To avoid interference from external stray light, and to assemble the optical and electronic components, we utilized 3D-modeling software (Rhino v8.0, Seattle, WA, USA) to design the casing and internal structure of the prototype platform. The components were meticulously printed using high-quality black ABS (acrylonitrile butadiene styrene) copolymers, ensuring precision and durability. Additionally, we designed and printed fixtures to mount the OPU, fluorescence-detection module, stepping motor, and test strips. The left chamber is responsible for placing the stepping motor and the sample, and the right chamber is used to fix the electronic module. There is a hole in the casing for inserting test strips, and the final results will be displayed on the liquid crystal display (LCD). The button and switch are responsible for turning on/off the system and the motion of the motor, respectively. Figure 3D shows the 3D rendering of the overall appearance of our prototype platform, which weighs 0.52 kg and measures 151 × 11.2 × 10.8 mm^3^ (length × width × height).

#### 2.3.3. Electronic Module

The schematic diagram of the electronic module is presented in Figure S1, with all of the electronic components seamlessly integrated into a specially designed and printed circuit board. When the laser in the OPU is focused on the T-line or-C line of the ICTS, fluorescence is instantaneously excited. In high concentrations, fluorescence becomes distinctly visible to the naked eye. It is worth noting that users should be cautious when operating the OPU laser. Once the fluorescence emitted by the test strip is detected by the photodiode, the fluorescence signal is converted into a voltage signal and amplified through a trans-impedance amplification circuit. At this point, numerous sources of noise are also amplified synchronously. To filter out the majority of the noise interference, the signal is passed through a high-order low-pass filter. Subsequently, the purified fluorescence signal is then transformed into a digital signal by a precision 16-bit analog-to-digital converter (ADC, ADS1115, Texas Instruments, Dallas, TX, USA), ensuring accurate and reliable data conversion. Finally, the signal is collected and processed by the microcontroller unit (MCU, Arduino Nano, Turin, Italy) using the serial peripheral interface (SPI) communication protocol. The entire platform is powered solely by an alkaline battery supplied by Nanfu Co., Ltd. (Nanping, China), eliminating the need for any additional power sources and enabling standalone operation.

#### 2.3.4. Detection Procedure

The MCU outputs PWM signals to control the movement of the stepping motor using a motor driver. As the stepping motor carries the test-strip movement at a distance of 0.1 mm between each readout point, the focused laser begins scanning until the fluorescence values of the T-line and C-line are collected. The laser is synchronized with the stepping motor, ensuring precise correspondence between the positions of the T-line and C-line with the data points programmed into the system. To overcome the error caused by the uneven chromatography of QDFM, we utilized the lateral scanning mechanism of the VCM in the OPU. Multiple points on the T-line and C-line were then calculated as the average. After scanning is completed, the collected fluorescence value of the T-line is divided by the fluorescence value of the C-line to obtain the T/C ratio. Finally, this ratio is displayed on the LCD screen, thereby concluding the quantitative detection process and providing the user with pertinent information. The duration from sampling to the final results will not exceed 15 min.

### 2.4. Preparation of the QDFM-mAb Complex

We prepared the QDFM-mAb probe by conjugating antibodies to quantum-dot fluorescence microspheres [33]. First, to prepare the QDFM suspension, 10 μL of QDFM were mixed with MES solution (pH 6.0, 25 mM) and centrifuged at 15,000 rpm for 10 min to suspend it in 100 μL of MES solution. Then, 2 μL of EDC (10 mg/mL) and 5 μL of NHS (10 mg/mL) were added to the mixture, followed by incubation in the dark at 25 °C for 20 min. After purification, the activated QDFM was mixed with 1 mL of PB solution (10 mM, pH 7.7) and FluA detection antibody, then incubated for 1.5 h. Subsequently, to block unreacted binding sites on the QDFM, 100 μL of a 20% BSA solution were introduced. The conjugate was then centrifuged at 15,000 rpm for 8 min. The final conjugate was resuspended in 100 μL of PBS buffer (10 mM, pH 7.4) containing 10% sucrose, 5% trehalose, 1% BSA, and 1% Tween-20, eventually being stored at 4 °C for further utilization.

### 2.5. Assembly of the Standard FluA ICTS

During the preparation of the ICTS, two NC membranes were adhered to a PVC bottom plate. Subsequently, FluA-capture antibody and goat anti-mouse IgG were sprayed onto the designated test line (T-line) and control line (C-line), respectively. The plates were then placed in a dry box maintained at a temperature of 37 °C for a duration of three hours. After this drying process, the sample pad, absorbent pad, and conjugate pad were assembled onto the PVC bottom plate. The plates were then cut into strips using a cutting machine. Finally, the ICTS were stored in a controlled drying atmosphere maintained at 37 °C, ready for future utilization.

### 2.6. Reagents and Instruments

The water-soluble carboxylated ZnCdSe/ZnS QDFMs were purchased from JiaYuan Quantum Dot Technology Co., Ltd. (Wuhan, China). The FluA recombinant antigen and monoclonal antibody were obtained from Yuduo Biotechnology Co., Ltd. (Shanghai, China). Bovine serum albumin (BSA), trehalose, and sucrose were bought from China National Pharmaceutical Group Corporation. Goat anti-mouse IgG was purchased from Solarbio Science & Technology Co., Ltd. (Beijing, China). N-hydroxysuccinimide (NHS), 1-ethyl-(3-dimethylaminopropyl) carbodiimide hydrochloride (EDC), and 2-morpholinoethanesulfonic acid (MES) were purchased from Aladdin Bio-Chem Technology Co., Ltd. (Shanghai, China). Phosphate-buffered saline (PBS) was purchased from Ser-Vicebio Science & Technology Co., Ltd. (Beijing, China). Nitrocellulose membrane (NC membrane, CN140) was purchased from Sartorius (Shanghai, China). The sample pad and conjugate pad were purchased from Jieyi Biotechnology Co., Ltd. (Shanghai, China), and the absorbent pad and PVC baseplate were obtained by Weice Biotechnology Co., Ltd. (Nanjing, China).

We utilized a commercial XYZ three-dimensional stripe-coating and gold-spraying instrument from Jinbiao Biotechnology Co., Ltd. (Shanghai, China) to uniformly spray antibodies onto the T-line and C-line. The fluorescence excitation and emission spectra of the QDFMs were measured by a spectrometer from Spark Tecan (Männedorf, Switzerland). The spectrum of the laser diode in the OPU was acquired by a spectrometer from Ocean Optics (Largo, FL, USA). The transmittance of the emission filter was obtained by a spectrometer from Perkin Elmer Instruments Co., Ltd. (Waltham, MA, USA).

### 2.7. Data Acquisition

Utilizing the Arduino IDE 2.0 open-source software platform, a program was crafted employing a programming language similar to C++. The fluorescence data-acquisition rate is set at 10 Hz/s, ensuring swift yet accurate data collection. The gathered data will then be averaged to minimize potential errors and enhance the overall reliability of the results. 

## 3. Results

### 3.1. Performance Testing

**Figure 4:**
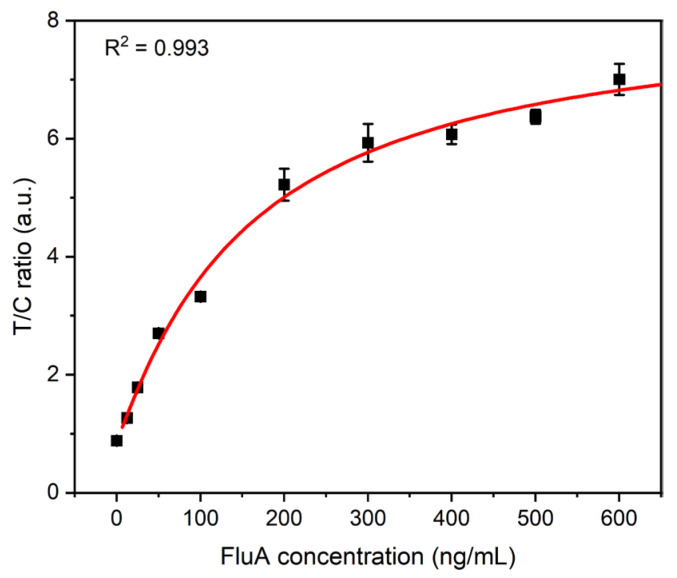
Results of FluA with different concentrations tested by the proposed platform. The error bars represent the standard deviation calculated from three replicated experiments.

To comprehensively evaluate the analytical performance of the fluorescence detection platform that we have developed, FluA recombinant antigens were diluted to different concentrations (0, 12.5, 25, 50, 100, 200, 300, 400, 500, and 600 ng/mL). The above-diluted solutions of different concentrations were mixed with the QDFM-mAb complex, respectively, and then dropped onto the ICTS sample pad. After 10 min of incubation, the strips were inserted into our proposed detection platform. All samples were prepared in a standardized dilution buffer to ensure consistency and reproducibility. Figure 4 clearly depicts the correlation between the fluorescence-intensity ratio of the T-line to the C-line and the varying concentrations of recombinant FluA antigen. The red line serves as the standard curve, facilitating the quantitative detection of FluA. As the concentration increases, a greater number of antigens are captured on the T-line, whereas the fluorescence intensity on the C-line remains relatively consistent. This results in an elevation of the T/C ratio. The coefficient of the correlation (R^2^ = 0.993) value underscores the existence of a robust correlation, indicating the accuracy of the system. Furthermore, the limit of detection (LOD), determined through a sigmoidal curve fit, represents the concentration of FluA at which the intensity value surpasses three times the standard deviation (SD) of the blank measurements, providing a quantitative threshold for detecting the presence of FluA. It was found to be 2.91 ng/mL, underscoring the low-cost system’s sensitivity in detecting low concentrations of FluA.

### 3.2. Specificity Testing

**Figure 5:**
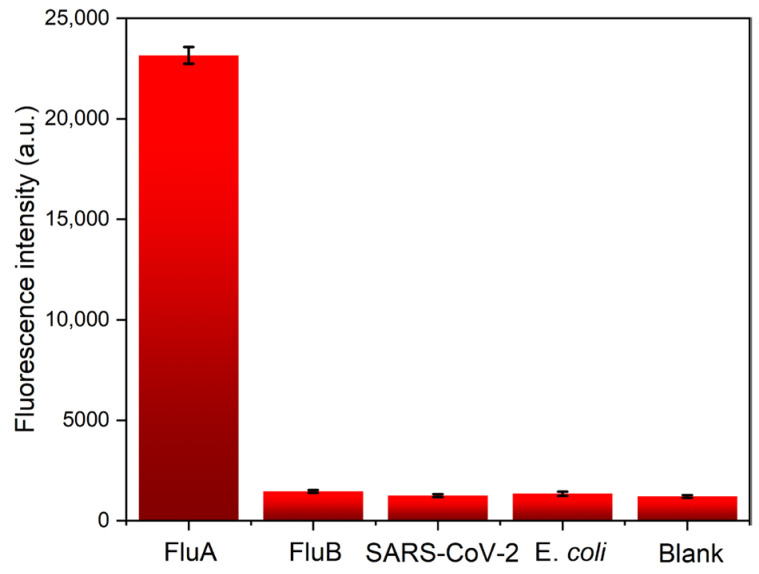
The specificity of five kinds of ICTS by the proposed platform. The concentrations of FluA, FluB, SARS-CoV-2, and *E. coli* are all 1000 ng/mL. The error bars represent the standard deviation calculated from three replicated experiments.

To validate the practical utility of our fluorescence-detection platform, we conducted specificity testing. As illustrated in Figure 5, when tested against high concentrations (1000 ng/mL) of FluA, FluB, SARS-CoV-2, *E. coli*, and a blank control, negligible signals were observed. However, the FluA ICTS displayed a robust signal, demonstrating the assay’s specificity towards the target analyte. These results confirm the reliability and effectiveness of our detection platform in practical applications.

### 3.3. Repeatability and Stability Testing

**Figure 6:**
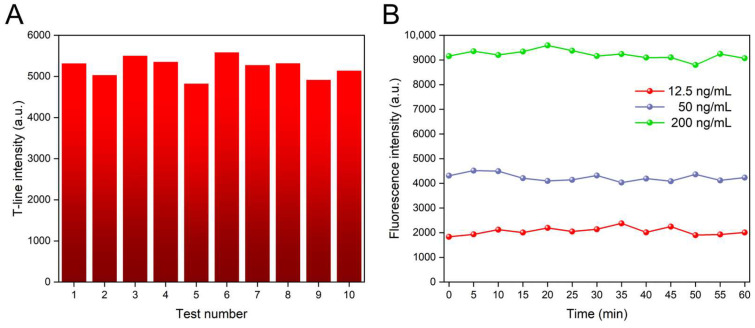
(**A**) Repeatability test of the proposed platform. (**B**) Stability test of the proposed platform.

The reproducibility of the signal was investigated to assess its reliability and consistency across different experimental conditions, as shown in Figure 6A. The coefficient of variation (CV) of 10 independent tests of FluA (100 ng/mL) was calculated at 4.69%, indicating good signal reproducibility. Furthermore, to investigate the signal stability of the proposed platform, we further tested the device to examine its performance over an extended period of time. We inserted ICTS with concentrations of 12.5 ng/mL, 50 ng/mL, and 200 ng/mL into the platform and recorded the T-line fluorescence values every five minutes for one hour. Figure 6B shows that the CV in fluorescence signals for concentrations of 12.5 ng/mL, 50 ng/mL, and 200 ng/mL were 2.06%, 3.61%, and 7.43%, respectively. The ten measured points were from different locations. Although higher antigen concentrations exhibited greater signal variation, the overall stability of our device during the testing process was demonstrated.

## 4. Conclusions

In conclusion, we have developed a lateral flow chromatographic immunoassay platform based on OPU for the rapid quantitative detection of FluA by lateral flow immunoassay test strips (ICTS). Through the integration of 3D-printed components, miniaturized optical elements, and customized electronic control boards, we have successfully developed a portable, cost-effective LFIA platform. Our prototype platform exhibits the potential for rapid detection of infectious disease target analytes in vulnerable populations, owing to its sensitivity, specificity, and stability. This platform stands out as an accessible solution for a diverse user base, including non-professionals, students, and the elderly, due to its compact size, light weight, and low cost. Significantly, our work represents an effort in the field, as it marks the first reported instance of a portable LFIA detection platform using OPU for FluA detection. Furthermore, our proposed platform enables a seamless transition to detecting other types of fluorescent dyes by replacing the emission filter when the fluorescence excitation spectrum matches the three kinds of OPU laser diodes. Therefore, it is versatile, flexible, and able to cater to different research needs, especially within the realm of biomedicine. 

## Data Availability

Data are contained within the article.

## Supplementary Materials

The following supporting information can be downloaded at https://www.mdpi.com/article/10.3390/bios14060263/s1, Figure S1: Electronic module schematic of the proposed platform; Figure S2: The characterization results of QDFM and QDFM-mAb; Figure S3: The stability of QDFM suspension on the first and seventh day.
