# Supplementary material for: A Portable Fluorescent Lateral Flow Immunoassay Platform for Rapid Detection of FluA
Source: Biosensors (Basel). 2024 May 21;14(6):263. doi: 10.3390/bios14060263 (PMC11201603; doi:10.3390/bios14060263)
Supplement: Supplementary file 1 [file biosensors-14-00263-s001.zip › biosensors-2993034-supplementary.pdf]

biosensors

MDPI

Supplementary Material

A Portable Fluorescent Lateral Flow Immunoassay Platform for Rapid Detection of FluA

Xu Chen [1,2], Xuhui Huang [1,2], Saima Kanwal [1,2], Jian Wang [1,2], Jing Wen [1,2,*] and Dawei Zhang [1,2]

[1] School of Optical-Electrical and Computer Engineering, University of Shanghai for Science and Technology, Shanghai, 200093, China
[2] Engineering Research Center of Optical Instrument and System, The Ministry of Education, Shanghai Key Laboratory of Modern Optical System, University of Shanghai for Science and Technology, Shanghai, 200093, China
* Correspondence: jwen@usst.edu.cn

1. The Control Methods for the Laser Diodes in the OPU

The laser control in the OPU features two methods. On one hand, the OPU has a built-in laser driver, model ATR0885, which allows users to define the laser wavelength and power by setting the electrical levels of the FFC connector. In the case of controlling the laser, people should set a high level (5 V) to pins 9, 10, 11, and 13, and ground to pins 7, 17, 19, and 21. The laser wavelength is selected by a 2-bit digital selector (pins 15 and 16) built in the OPU: 00 represents laser off; 11 represents a 405 nm laser; 01 represents a 650 nm laser; 10 represents a 780 nm laser. Additionally, the laser power can be defined by using pulse width modulation (PWM) to set the intensity bias via pin 18. The power of the laser will be controlled by an 8-bit PWM signal, which corresponds to a voltage range of 0-5V. On the other hand, the laser diode built into the OPU can also be controlled by an external DC power source, simply by identifying and connecting to the corresponding positive and negative terminals of the laser diode. The relevant parameters of the 405nm laser diode are as follows:

(1) Blue–violet laser diode: manufactured by SHARP (Japan), and the model number is GH04P21A2GE;

(2) Wavelength: 406 nm (Typ.), 400 nm (Min), 413 nm (Max);

(3) Optical power output: CW 105 mW (Max)
Pulse 210 mW (Max);

(4) Operating current: 120 – 150 mA

(5) Operating voltage: 5.4 – 6.5 V;

(6) Operating temperature: -10℃ – 70℃.

2. Electronic Module Schematic

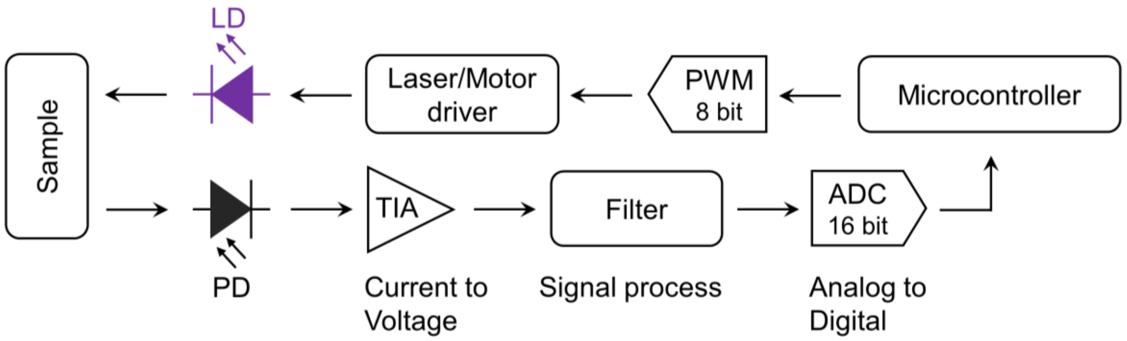

Figure S1. Electronic module schematic of the proposed platform.

3. Characterization Results of the QDFM

Since we used the QDFM-mAb for LFIA fluorescence detection, it is important to characterize the properties of the QDFM itself. First, we used transmission electron microscopy (TEM) to characterize the appearance of the QDFM. As shown in Figure S2(A, B), clusters of multiple quantum dots (QDs) are encapsulated as dark spots in a polymer shell to prepare QDFM. The average size of the QDFMs is approximately 100 nm. In addition, we also measured the fluorescence excitation and emission spectra of the QDFM and QDFM-mAb probes. As shown in Figure S2(C), both QDFM and QDFM-mAb have an excitation peak at 365 nm. Considering that the laser wavelength we used is 405 nm, they also have a relatively high excitation efficiency at 405 nm. As shown in Figure S2(D), both QDFM and QDFM-mAb display fluorescence emission peaks near 625 nm. As depicted in Figure S2(C,D), the consistency in fluorescence excitation and emission peaks between QDFM and QDFM-mAb underscores the effective coupling of the QDFM with the monoclonal antibody, affirming its suitability for the quantitative detection of FluA. The QDFM suspension remained stable for a week, which can be observed in Figure S3.

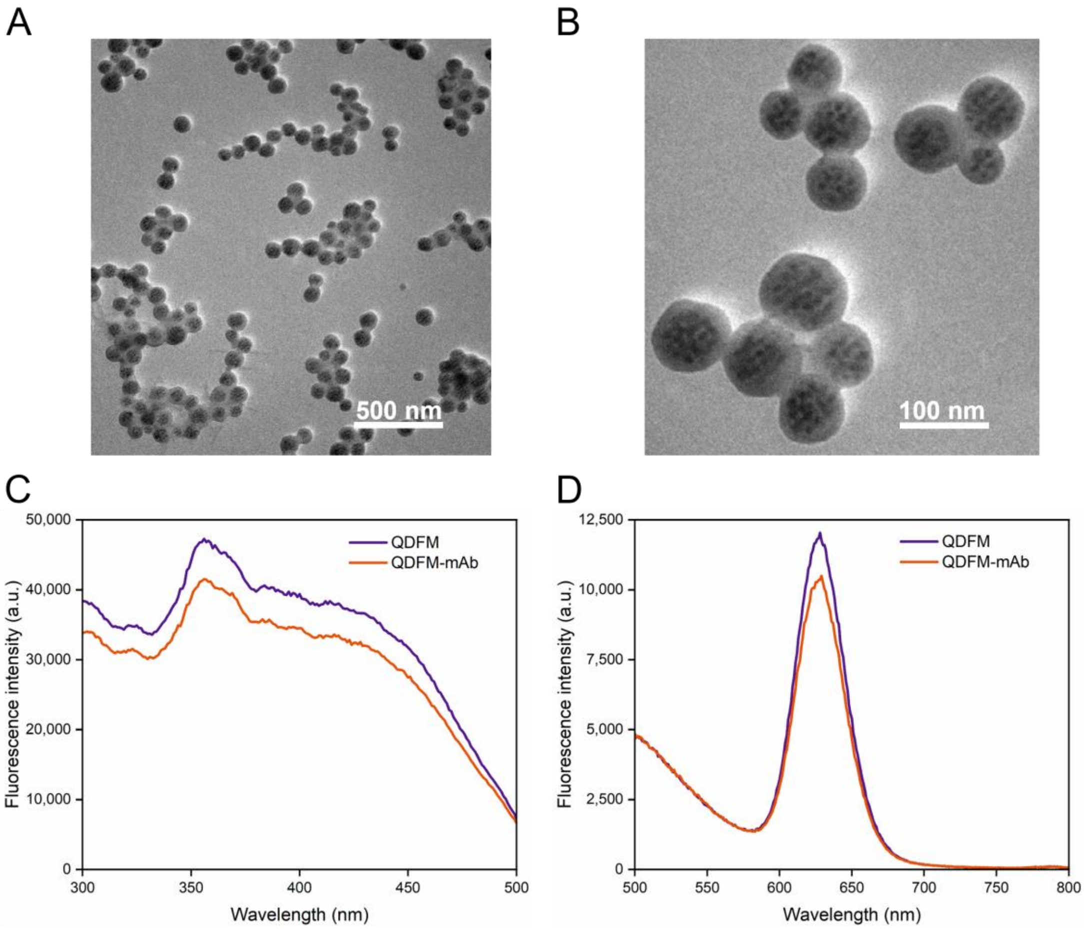

Figure S2. The characterization results of QDFM and QDFM-mAb are presented as follows: (A, B) TEM images of QDFM clusters captured at varying magnifications and (C, D) fluorescence excitation and emission spectra of QDFM and the prepared QDFM-mAb conjugate, respectively.

4. Stability of the QDFM

As can be seen from **Figure S3,** there was no significant change in color and precipitation of the QDFM suspension on both the 1st and 7th day, indicating the excellent colloidal stability of the suspension.

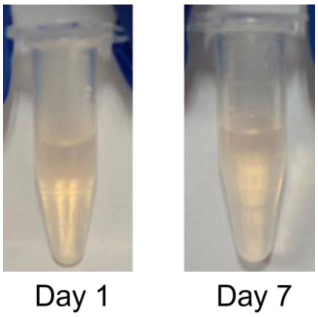

Figure S3. The stability of QDFM suspension on the first and seventh day.
